# Potential reversal of biological age in women following an 8-week methylation-supportive diet and lifestyle program: a case series

**DOI:** 10.18632/aging.204602

**Published:** 2023-03-22

**Authors:** Kara N. Fitzgerald, Tish Campbell, Suzanne Makarem, Romilly Hodges

**Affiliations:** 1 Institute for Functional Medicine, Federal Way, WA 98003, USA; 2 Virginia Commonwealth University, Richmond, VA 23284, USA; 3 American Nutrition Association, Hinsdale, IL 60521, USA

**Keywords:** DNA methylation, epigenetic, aging, lifestyle, biological clock

## Abstract

Here we report on a case series of six women who completed a methylation-supportive diet and lifestyle program designed to impact DNA methylation and measures of biological aging. The intervention consisted of an 8-week program that included diet, sleep, exercise and relaxation guidance, supplemental probiotics and phytonutrients and nutritional coaching. DNA methylation and biological age analysis (Horvath DNAmAge clock (2013), normalized using the SeSAMe pipeline [a]) was conducted on blood samples at baseline and at the end of the 8-week period. Five of the six participants exhibited a biological age reduction of between 1.22 and 11.01 years from their baseline biological age. There was a statistically significant (p=.039) difference in the participants' mean biological age before (55.83 years) and after (51.23 years) the 8-week diet and lifestyle intervention, with an average decrease of 4.60 years. The average chronological age at the start of the program was 57.9 years and all but one participant had a biological age younger than their chronological age at the start of the program, suggesting that biological age changes were unrelated to disease improvement and instead might be attributed to underlying aging mechanisms.

## INTRODUCTION

Six in ten adults in the United States have at least one chronic disease and four in ten adults have two or more [1]. These diseases are a major cause of morbidity and mortality and they put a significant burden on the healthcare system as well as the society at large [2]. Aging itself has been identified as a common driver of chronic diseases and an important target for extending human healthspan [3]. It has also been estimated that if we improve our collective healthspan by just one year the calculated savings are worth $38 trillion dollars, and if by 10 years those savings jump to $367 trillion dollars [4]. Biological age clocks, based on DNA methylation marks, have become important surrogate markers to assess the effectiveness of interventions at reducing biological age, with the expectation that biological age reductions will compress morbidity and extend mortality [5, 6].

Modifiable lifestyle factors, including concentrated exposure to dietary “epinutrients”, have been suggested to be able to favorably influence DNA methylation-based clocks and therefore have the potential to compress morbidity and extend mortality [7]. Epinutrients may be defined as dietary nutrients that provide either substrates or cofactors for DNA methylation activity or influence the expression or rate of activity of DNA methylation-related enzymes. Folate and betaine, for example, are cofactors in methylation biosynthetic pathways, alpha ketoglutarate, vitamin C, and vitamin A are ten-eleven translocation (TET) demethylase cofactors and modulators, and curcumin, epigallocatechin gallate (EGCG), rosmarinic acid, quercetin, and luteolin are known polyphenolic modulators of DNA methyl transferase (DMNT) enzymes [8, 9].

The modifiable lifestyle intervention used by participants in this case series was first investigated in a pilot clinical trial in which participants (all men between the ages of 50-72 years) reduced their biological age by an average of 3.23 years as compared to controls [7]. The case series reported on herein was conducted to further the investigation of a modifiable lifestyle intervention that was largely the same in other populations; importantly in women.

## RESULTS

### Participants’ biological age change

Five of the six participants that followed the program saw a reduction in biological age, while one participant saw no change (Table 1) [7]. Of those six, the maximum reduction in biological age was 11.01 years, while the minimum reduction in biological age was 1.22 years, with a mean biological age reduction of 4.6 years.

**Table 1:** Participants’ biological age at baseline and endpoint.

**Participant**	**Chronological age**	**BioAge_baseline_**	**BioAge_endpoint_**	**Change**
1	62	57.33	46.32	-11.01
2	56	53.85	49.78	-4.07
3	56	52.79	45.39	-7.40
4	65	61.63	60.41	-1.22
5	62	60.30	60.30	0
6	46	49.09	45.15	-3.94

**Figure 1:**
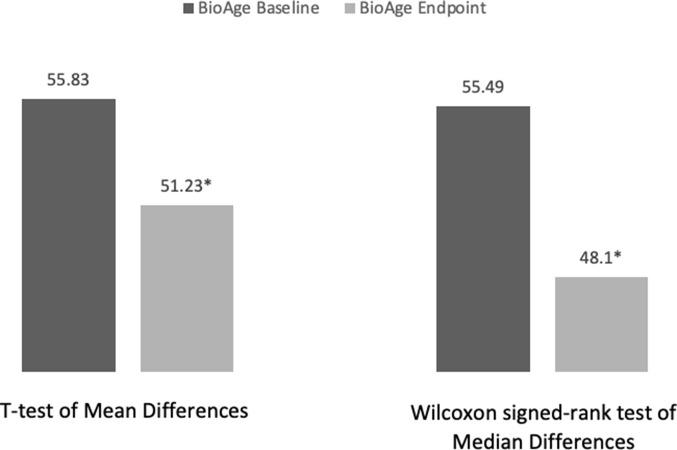
**Participants’ average biological age change analysis.** *Significant difference with p-value < .05.

A paired sample t-test was conducted to compare the biological age of the participants at baseline (BioAge_baseline_) and the biological age of the participants at the end of week 8 (BioAge_endpoint_). The results show a significant difference between mean baseline biological age (m_baseline_ = 51.23) and mean endpoint biological age (m_endpoint_ = 55.83) (t-statistic = 2.783, *p*-value = 0.039) (Figure 1). Given the small sample size, we conducted a Shapiro-Wilk test of normality and found that participants’ biological age at baseline (Shapiro-Wilk Statistic = .959, *p* = .814) is normally distributed. However, biological age at endpoint is not (Shapiro-Wilk Statistic = .775, *p* = .035). Therefore, we performed a non-parametric Wilcoxon signed-rank test to compare the median biological age at baseline to the media biological age at endpoint. The Wilcoxon signed-rank test showed that the diet and lifestyle intervention elicited a significant change in the biological age of participants (Z = -2.023; *p* = .043) with the median biological age at baseline = 55.49 being significantly higher than the median biological age at the end of the 8-week intervention = 48.1 (Figure 1).

### Participant adherence to the program

For the diet and lifestyle goals listed in Table 2, participants checked a box to “Mark as done” if they met the listed goal. Over the 8 weeks of the study, data reporting was relatively high with an average of 6.44 days reported per week across all six participants. Adherence for each goal was calculated based on the percentage of days reported that a participant checked a goal. Average individual adherence across all goals varied between 71.2% and 97.2%, with an average of 81.7%. Average adherence according to week had less variance, with the lowest adherence average across participants observed for week 8 (76.0%) and highest such adherence observed at week 6 (86.7%). For the six participants, average individual adherence to diet and supplements goals (84.4%) seems to have been easier than adherence to lifestyle goals (77.0%), with the breathing exercise being most challenging (average adherence-breathing exercise = 57.5%) and intermittent fasting being least challenging (average adherence-intermittent fasting = 93.7%).

**Table 2:** Summary of dietary and lifestyle interventions delivered through the app.

**“Today’s foods”**	**“Today’s supplements”**
2 cups, dark leafy greens 2 cups cruciferous vegetables 3 cups colorful vegetables ¼ cup pumpkin seeds ¼ cup sunflower seeds 2 servings methylation adaptogens 1 to 2 beets Liver or liver supplement (three, 3-ounce servings per week) 1 serving egg (5-10 per week)	Probiotics (2 capsules) Greens powder (*2)
**“Healthy habits daily recap”**	**“Water goal”**
I exercised for at least 30 minutes I practiced breathing exercises twice I slept at least 7 hours I fasted 12 hours after my meal	8 cups per day

## DISCUSSION

### Significance of biological age change

The findings of this case series of 6 women following the methylation-supportive diet and lifestyle program include changes to DNA methylation patterns leading to an average reversal of epigenetic biological age of 4.60 years with a statistically significant difference between baseline and endpoint biological age (p=.039). The original pilot study of a dietary and lifestyle program that was largely the same as this also resulted in an average reversal of biological age of 3.23 years as compared to controls (p=.018), accounting for any normal variability in epigenetic methylation [7]. Both studies used the same Horvath DNAmAge clock (2013) to measure biological age at baseline and the end of the intervention period. Participants in this case series were all women, between the ages of 45 and 65, unlike the original pilot cohort which were men between the ages of 50-72. Taken together, these data suggest that a methylation-supportive diet and lifestyle intervention may favorably influence biological age in both sexes during middle age and older.

### Participant adherence to the intervention

As suggested by analysis of adherence data in this case series, 100% adherence to the program is not required to achieve positive outcomes, since the improvements in biological age were observed with goals-based adherence of between 71.2% and 97.2% (average 81.7%). We attribute the relatively high participant adherence in this case series in part to the nutrition coaching support provided. The level of adherence is notable given participants engaged in the program during the months of November through January, a time when many Americans celebrate food-centric holidays, and adherence to dietary programs can be more challenging.

### Significance of studying healthy individuals

Studying individuals who are considered generally healthy at baseline, as is the case in this case series as well as the original pilot study, may have removed potential confounding from disease-accelerated biological aging and instead limited the investigation to the influence of the intervention on aging itself. It has been documented that those with chronic disease, such as type 2 diabetes, have epigenetic methylation patterns that are consistent with being biologically older than their disease-free counterparts, and that those with a higher biological age who are presently disease-free are at greater risk of developing a chronic disease [10, 11]. In addition, it has been observed in large data sets that positive lifestyle factors as well as effective medical treatment correlate with a reduced biological age in those with type 2 diabetes (Preprint) [11]. The participants in the original pilot study of this intervention were considered to be healthy as defined by an exclusion of diagnosed illness [7]. In this case series, five (out of six) participants may be considered healthy as is suggested by their baseline biological age being less than their chronological age [12].

### One participant who did not complete the program

There was a seventh, male participant in this case series who started the intervention, but who dropped out before completing the program due to a reported family emergency. As such he was not eligible to be included in the final analysis. His baseline DNAmAge was 57.59 and chronological age was 71. Even though he did not complete the program, he did obtain his DNAmAge at the eight-week time point, where it had increased to 61.58. Sudden acute acceleration in biological age due to diverse stressful events (which is then reversed following recovery from the event) has been documented (Preprint) [13].

## CONCLUSIONS

The findings of this case series add to the existing evidence suggesting that widely-accessible, cost-effective dietary and lifestyle interventions, that are designed to support DNA methylation and are widely considered to be safe, may be able to reduce measures of biological aging and have the potential to impact healthspan, lifespan, and the economic burden of aging. This case series of women participants extends the previous pilot study of this intervention in men, indicating that favorable biological age changes may be achievable in both sexes. In addition, the investigation of otherwise-healthy individuals, rather than those with diagnosed disease, suggests an influence directly on underlying mechanisms of aging instead of disease-driven aging.

A significant limitation of this case series is its small cohort size. Other limitations include its use of a biological age assessment based on the first-generation Horvath multi-tissue clock and the lack of a control group that allows for ruling out changes in biological age due to factors outside the intervention. Future research will need to include larger, more diverse cohorts, the use of more advanced clocks and a control group. Such future research should also examine the relationship between diet and lifestyle program adherence and biological age change.

## MATERIALS AND METHODS

### Study participants

The six participants included in the final analysis of this study, all female between the ages of 46 and 65, were the first and only individuals to complete the 8-week diet and lifestyle program and obtain comparable baseline and endpoint biological age testing as measured by the Horvath DNAmAge clock. Recruitment of study participants was conducted via advertisement on various websites and social media platforms maintained by Dr. Kara Fitzgerald and participants completed the program between November 2021 and January 2022.

After the 7th case (the individual referenced above who stopped following the program before completion), due to change in laboratory methodology, we were unable to obtain further comparable Horvath DNAmAge data from additional participants. Therefore, within the constraints of comparable data on the Horvath DNAmAge clock at baseline and endpoint, no cases were omitted.

### Study design

Participants followed an intervention that included a specific set of dietary recommendations high in known epinutrients. Simple carbohydrates were restricted, and the diet was largely plant centered but included key nutrient-dense animal protein from 5-10 eggs per week, 6 oz of animal protein daily, and three 3-ounce servings of liver per week (or an encapsulated liver supplement). Participants were also asked to eat all food within a 12-hour window each day to incorporate a basic level of intermittent fasting. Different from the original study, participants were encouraged to track their water consumption aiming for 8-cups of water per day.

Dietary supplements consisted of a probiotic containing 40 million CFU of Lactobacillus plantarum 299v. L. plantarum (UltraFlora® Intensive Care, Metagenics Inc. Aliso Viejo, CA, USA) and a fruit and vegetable powder, rich in additional polyphenolic compounds, twice a day (PhytoGanix®, Metagenics Inc. Aliso Viejo, CA, USA).

Lifestyle modifications that participants were asked to incorporate included a minimum of 30 minutes of physical activity at least 5 days a week, at an intensity of 60-80 percent of maximum perceived exertion. All participants were encouraged to get a minimum of seven hours of sleep per night, participate in two, 10-minute breathing sessions per day designed to elicit the relaxation response (a meditation video was provided).

The intervention program was delivered through the beta testing of a HIPAA-compliant digital application [herein the app]. The app provided video and written instruction, daily tracking tools, optional recipes, a shopping list, and reminders for participants to complete. A summary of dietary and lifestyle interventions delivered through the app, displayed as “Today’s Foods”, “Today’s Supplements”, “Water Goal”, and “Healthy Habits Daily Recap” are shown in Table 2. An overview of the intervention is shown in Table 3.

**Table 3:** Overview of the dietary and lifestyle intervention*.

**Intervention category**	**Details**
Dietary Prescription	*Guidance per week:*
	**3 servings of liver** (1 serving = 3 oz) Preferably organic
	**5-10 eggs** Ideally free-range, organic, omega-3 enriched

	*Guidance per day:*
	**2 cups of dark leafy greens** Measured raw, chopped, and packed Including kale, Swiss chard, collards, spinach, dandelion, mustard greens Does not include salad greens such as romaine, iceberg, Spring mix
	**2 cups cruciferous vegetables** Measured raw, chopped, and packed Includes broccoli, cabbage, cauliflower, Brussels sprouts, bok choy, arugula, kale, mustard greens, watercress, rutabaga, kohlrabi, radish, Swiss chard, turnip
	**3 additional cups colorful vegetables** of your choosing (excluding white potatoes, sweetcorn)
	**1-2 medium beet**
	**4 tbsp (1/4 cup) pumpkin seeds** (or pumpkin seed butter)
	**4 tbsp (1/4 cup) sunflower seeds** (or sunflower seed butter)
	**1+ serving methylation adaptogens, choose from:** 1/2 cup berries (wild preferred) 1/2 tsp rosemary 1/2 tsp turmeric 2 medium cloves garlic 2 cups green tea (brewed 10 minutes) 3 cups oolong tea (brewed 10 minutes)
	**6 oz animal protein** Grass-fed, pastured, organic and hormone/antibiotic-free
	**2 servings of low glycemic fruit**
	*General guidance:*
	**Organic** preferred over conventional
	**Stay hydrated**
	**Don’t eat between** 7pm and 7am
	**Include “healthy” oils** Balance types of fat E.g. coconut, olive, flaxseed and pumpkin seed oil
	**Avoid** added sugar/candy, dairy, grains, legumes/beans
	**Minimize** plastic food containers
Supplement Prescription	PhytoGanix®, a combination of organic vegetables, fruits, seeds, herbs, plant enzymes, prebiotics and probiotics at a dose of 2 servings daily, divided UltraFlora® Intensive Care, containing *Lactobacillus plantarum 299v* at a dose of 2 capsules daily, divided
Exercise Prescription	Minimum of 30 minutes of exercise per day for at least 5 days per week, at an intensity of 60-80% of maximum perceived exertion
Sleep Prescription	Average a minimum of 7 hours of sleep per night
Stress Management Prescription ^A^	Breathing exercise *Steps to Elicit the Relaxation Response* developed by Herbert Benson MD, twice daily

^A^Stress Management Recommendations were updated from the original Study Protocol as listed on ClinicalTrials.gov. All updates were IRB approved. *Patent pending.

As in the original pilot study, adherence to the program was supported by regular coaching sessions conducted by trained nutritionists, delivered weekly during the first four weeks, and then at least every other week thereafter. Nutrition coaches had a predefined list of questions that covered adherence to intervention guidelines and queried any changes to medications. Participants had access to nutrition coaches between sessions via texting within the app, with an expected response time of 24-48 hours.

### Laboratory testing and DNAmAge analysis

Biological age testing was conducted at baseline and at week 8 for all six participants by TruDiagnostc Laboratory. To calculate the Horvath DNAmAge clock, the agep() function in the wateRmelon R package was used. Beta values were normalized using the SeSAMe pipeline [a].

For consistency with the first pilot study of this diet and lifestyle intervention, this case series also used Horvath’s original DNAmAge Clock (2013), also known as the Pan-Tissue clock, which continues to be widely accepted, even as second- and third- generation clocks have since been developed. While it was trained against chronological age, multiple studies have shown acceleration of this clock to be associated with age-related disease risk and all-cause mortality [8].
